# Research on Fault Type Identification for Distribution Networks with Distributed Power Sources Based on Improved CNN-BiGRU

**DOI:** 10.3390/s26123947

**Published:** 2026-06-21

**Authors:** Lei Li, Weili Wu

**Affiliations:** College of Electrical and Control Engineering, Xi’an University of Science and Technology, Xi’an 710054, China; xkd_ll286@163.com

**Keywords:** distributed generation, distribution network, fault type identification, wavelet packet transform, CNN-BiGRU-Attention

## Abstract

The integration of distributed generation (DG) changes the fault current path, magnitude, direction, and transient characteristics of distribution networks, which increases the difficulty of fault type identification. In particular, weak fault features and high-frequency transient components may reduce the reliability of traditional feature-based diagnosis methods. To improve the representation and classification capability of fault signals, this paper proposes a fault type identification method based on wavelet packet transform and an improved CNN-BiGRU model with a channel attention mechanism. First, three-phase voltage, three-phase current, and zero-sequence voltage signals are decomposed by wavelet packet transform, and the corresponding time–frequency matrices are constructed. Then, these matrices are integrated and converted into time-frequency images, so that multi-source fault information can be represented in a unified form. On this basis, CNN is used to extract local spatial features from the time-frequency images, while BiGRU is employed to capture bidirectional dependency information of fault features. Furthermore, a channel attention mechanism is introduced to enhance informative feature channels and suppress redundant information, thereby improving the fault classification performance. Simulation results based on a 10 kV DG-integrated distribution network show that the proposed method achieves high recognition accuracy under different DG capacities and access configurations. Compared with CNN, BiGRU, and CNN-BiGRU models, the proposed CNN-BiGRU-Attention model shows better classification accuracy and adaptability, demonstrating its effectiveness for fault type identification in active distribution networks.

## 1. Introduction

With the rapid development of distribution systems and the large-scale integration of distributed generation (DG), the topology and power flow direction of conventional distribution networks have changed significantly. DG integration changes the fault current path, magnitude, and direction, and may increase the risk of protection miscoordination or maloperation [1,2,3]. Meanwhile, fault characteristics in active distribution networks are significantly different from those in traditional passive distribution networks. Fault signals often exhibit weak steady-state features, complex transient components, and obvious high-frequency variations. These factors increase the difficulty of fault detection and fault type identification and may affect the stable operation of distribution networks. Therefore, accurate fault type identification for DG-containing distribution networks is very important for the development of modern power systems [4,5,6,7].

In DG-integrated distribution networks, the non-stationary and nonlinear characteristics of fault signals mainly originate from abrupt changes in network topology, transient electromagnetic processes at the fault inception instant, and the control characteristics of DG units. After a fault occurs, voltage and current signals no longer remain ideal steady-state sinusoidal waveforms, but contain decaying DC components, high-frequency oscillatory components, transient impulse components, and sudden variations in amplitude and phase. In non-effectively grounded systems, transient grounding current is also jointly affected by line-to-ground capacitance, the arc suppression coil, transition resistance, and fault inception angle. After DG is connected, the system changes from a single-source radial structure to a multi-source active network. The access location, installed capacity, control strategy, and current-limiting characteristics of DG further change the magnitude, direction, and phase relationship of fault currents. As a result, the same fault type may present different waveform characteristics under different operating conditions, while different fault types may show similar features. Traditional methods based on fixed thresholds or single signal features may therefore suffer from insufficient robustness and poor adaptability. More specifically, these non-stationary and nonlinear components affect fault identification in three aspects. First, the transient components generated at the fault inception instant change rapidly with time, which makes the fault features extracted from a fixed time window sensitive to the fault inception angle, transition resistance, and sampling interval. Second, high-frequency oscillatory components and decaying DC components may weaken or distort the steady-state amplitude and phase relationships among three-phase voltage, three-phase current, and zero-sequence voltage. As a result, the discriminative features of different fault types become less stable. Third, after DG integration, the fault current contribution is affected by the DG access location, installed capacity, and current-limiting control strategy. This may cause the same fault type to exhibit different waveform characteristics under different DG configurations, while different fault types may show similar feature distributions. Therefore, the intra-class difference in fault samples increases, and the inter-class separability among fault types decreases, which increases the difficulty of fault identification. It is thus necessary to develop an adaptive fault identification method that can simultaneously represent low-frequency steady-state information and high-frequency transient information. 

At present, fault identification methods for traditional distribution networks mainly include steady-state characteristic analysis and transient characteristic analysis [8,9,10]. Steady-state methods usually determine the fault phase by analyzing phase-angle changes and amplitude distortion of three-phase voltages and currents. For example, Qi et al. [11] proposed an improved S-transform method and used harmonic content as the fault criterion. Wang et al. [12] adopted improved empirical mode decomposition to extract sudden singular points and cumulative slopes of zero-mode current for high-impedance fault identification. Wang et al. [13] performed preliminary discrimination based on modal current amplitude deviation and further identified fault types using gray relational analysis. These methods have certain effectiveness, but they usually rely on manually determined thresholds, and feature extraction becomes difficult when fault features are weak. In addition to the above methods, many studies have combined transient signal analysis with artificial intelligence algorithms for fault identification. For example, wavelet analysis has been used to extract singular spectrum entropy, fuzzy entropy, and composite multiscale permutation entropy from voltage or current signals, which are then combined with support vector machines for fault classification [14,15,16,17]. Time-frequency decomposition methods have also been used to construct two-dimensional matrices or images that describe the energy distribution of fault signals, and CNN-based models have been applied for automatic feature extraction and classification [18,19,20]. In addition, voltage–current characteristics, evidence information fusion, dual-channel neural networks, and the fusion of transient and steady-state information have been introduced to improve fault diagnosis performance. Wavelet coefficients of zero-sequence current and random forest algorithms have also been applied to grounding fault identification [21]. These studies have made important progress in extracting non-stationary transient features. However, under DG-integrated conditions, fault characteristics are further affected by DG access location, capacity, quantity, fault distance, and transition resistance, making the coupling relationship among multiple electrical quantities more complex. Therefore, it is still necessary to construct a unified fault representation that integrates three-phase voltage, three-phase current, and zero-sequence voltage information, and to develop an adaptive identification model suitable for different DG access scenarios.

Moreover, recent studies have shown that diagnostic information acquisition and sensor configuration have an important influence on fault diagnosis performance. For example, the optimal sensor placement methodology proposed for hydraulic control systems provides a useful reference for improving the observability and reliability of diagnosis systems [22]. Although its research object is different from the DG-integrated distribution network considered in this paper, it indicates that the quality and configuration of diagnostic information directly affect fault identification accuracy.

Although wavelet-based time-frequency analysis, CNN-based feature extraction, recurrent neural networks, and attention mechanisms have been applied in fault diagnosis, the novelty of this paper does not lie in treating these methods as independent new algorithms. Instead, the main innovation lies in constructing a unified fault identification framework that integrates multi-source time-frequency representation, spatial feature extraction, temporal dependency learning, and adaptive feature weighting for DG-integrated distribution networks. The specific innovations are summarized as follows. First, three-phase voltage, three-phase current, and zero-sequence voltage are jointly used to construct a composite fault representation, which provides richer fault information than methods based on a single electrical quantity. Second, wavelet packet decomposition is used to generate composite time-frequency images, so that both low-frequency steady-state components and high-frequency transient components can be retained. Third, CNN, BiGRU, and channel attention are organically combined: CNN extracts local spatial features from time-frequency images, BiGRU captures bidirectional temporal dependency information, and the channel attention mechanism enhances key feature channels. Therefore, the proposed method improves the adaptability of fault type identification under different DG access locations, capacities, and quantities.

Based on the above analysis, this paper proposes a fault type identification method for DG-integrated distribution networks. First, the wavelet packet transform is used to decompose three-phase voltage, three-phase current, and zero-sequence voltage signals, and time–frequency matrices are constructed in ascending order of frequency. Then, the matrices corresponding to the three types of electrical quantities are fused to form a composite time–frequency matrix, which is further converted into a time-frequency image as the model input. Finally, the time-frequency image is fed into the improved CNN-BiGRU model, and a channel attention mechanism is embedded in the fully connected layer of BiGRU to improve fault feature classification. Confusion matrices and t-SNE visualization are used to present the classification results. Experiments under different DG capacities and access quantities verify the adaptability and recognition accuracy of the proposed method.

## 2. Analysis of the Influence of Distributed Generation Access on Fault Characteristics

### 2.1. Analysis of Transient Fault Characteristics of Small Current Grounding Systems

**Figure 1:**
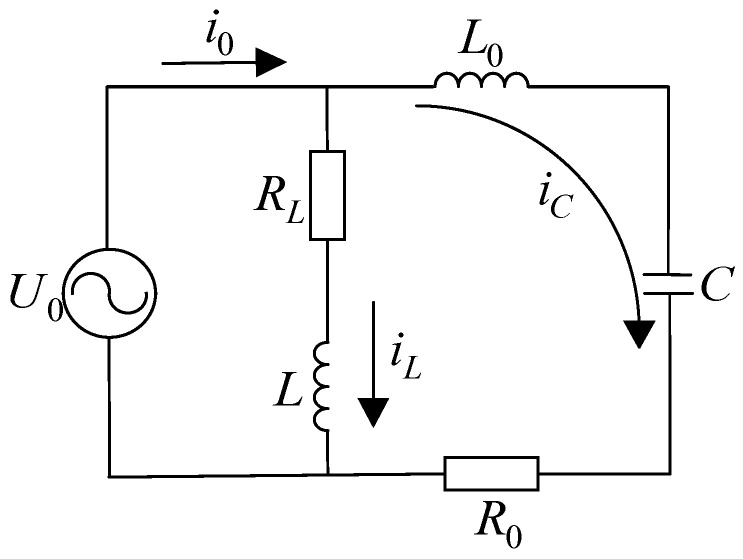
Equivalent circuit of single-phase ground transient current.

This section employs the Petersen coil grounding mode as a typical case. A transient equivalent circuit is constructed and depicted in Figure 1.

(1) Transient capacitive current

Based on Kirchhoff’s Voltage Law, the equation can be obtained:

(1) $$R_{0}i_{c}+L_{0}\frac{di_{c}}{d_{t}}+\frac{1}{C}\int i_{c}dt=U_{m}\sin(\omega t+\varphi )$$

where $U_{m}$ is the amplitude of zero-sequence voltage and φ is the initial phase angle of the fault. 

Transient capacitive current $i_{c}$ includes power-frequency steady-state component $i_{c\cdot ot}$ and high-frequency transient component $i_{c\cdot os}$.

(2) $$\begin{array}{l} i_{c}=i_{c\cdot ot}+i_{c\cdot os}=I_{cm}\cos\left(\omega t+\varphi \right)+\\ I_{cm}\left(\frac{\omega_{f}}{\omega }\sin\varphi -\cos\varphi \cos\omega_{f}t\right)e^{-\delta t} \end{array}$$

where $I_{cm}$ is capacitive current amplitude; $\omega_{f}$ is high-frequency transient angular frequency; δ is the free oscillation attenuation coefficient.

(2) Transient inductive current

According to Kirchhoff’s Voltage Law:

(3) $$U_{m}\sin(\omega t+\varphi )=R_{L}i_{L}+N\frac{d\phi_{L}}{dt}$$

where $\phi_{L}$ is the magnetic flux of the arc suppression coil core; N is coil turns.

Transient inductive current includes a transient DC component and a steady-state AC component:

(4) $$i_{L}=I_{Lm}\left[\cos\varphi e^{-\frac{t}{\tau_{L}}}-\cos(\omega t+\varphi )\right]$$

where $I_{Lm}$ is inductive current; $\tau_{L}$ is the time constant of the inductive loop.

(3) Transient ground fault current

The transient earth current $i_{d}$ is composed of the transient capacitive current and the transient inductive current in combination:

(5) $$\begin{array}{l} i_{d}=i_{c}+i_{L}\\ =I_{Lm}\cos\varphi e^{-\frac{t}{\tau_{L}}}+(I_{cm}-I_{Lm})\cos\left(\omega t+\varphi \right)\\ +I_{cm}\left(\frac{\omega_{f}}{\omega }\sin\varphi \sin\omega t-\cos\varphi \cos\omega_{f}t\right)e^{-\delta t} \end{array}$$

The first two items correspond to transient components. The third term represents the steady-state component. Its amplitude is determined by the difference between the steady-state capacitive current and inductive current.

### 2.2. Fault Characteristics of Distribution Networks with DG

**Figure 2:**
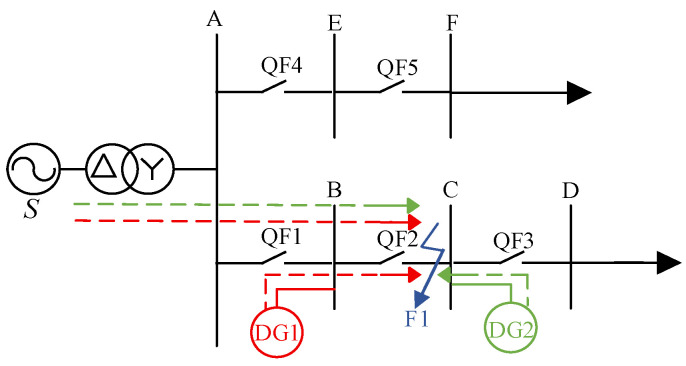
Power flow direction in a DG-equipped distribution network.

DG access to the distribution network leads to evident changes in network topology, operating state, and power flow direction. This paper investigates the transient fault behaviors of non-effectively grounded systems with DG connected at different sites. Figure 2 shows power flow directions under different DG access points. Studies show that DG position affects short-circuit current variation on system lines. DG positions are divided into two cases: (1) DG is placed at the upstream section relative to the fault location; (2) DG is installed at the downstream section relative to the fault location.

When DG1 is placed at point B and a fault occurs at F1, fault current is supplied by both the main system and DG1. This increases the current at QF2, which is called the current boosting effect. When DG2 is placed at point C and a fault occurs at F1, fault current is still supplied by the main system and DG2. However, it reduces the current at QF2, which is called the current drawing effect.

**Figure 3:**
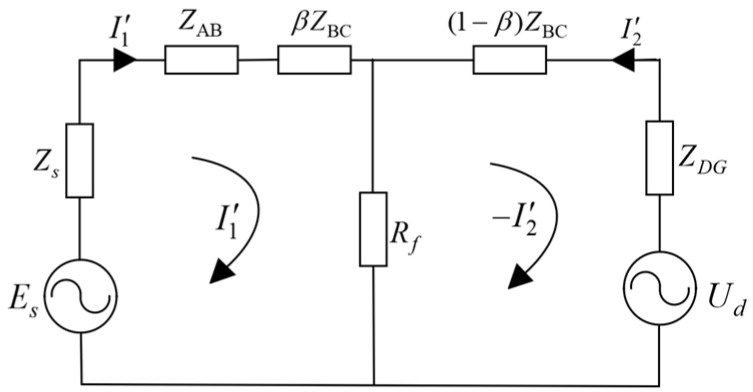
Equivalent Circuit of DG-Integrated Distribution Network.

The equivalent circuit with DG is shown in Figure 3, where $E_{s}$ is the system source electromotive force; $Z_{s}$ is the source impedance; $Z_{AB}$ and $Z_{BC}$ are the line impedances between points AB and BC, respectively; $Z_{DG}$ is the internal impedance of the distributed generation DG; $R_{f}$ is the fault resistance; *β* represents the ratio of the length from point B to the fault position over the total length of line BC.

Before DG integration

(6) $$I_{1}=\frac{E_{s}}{Z_{s}+Z_{\mathrm{AB}}+\beta Z_{BC}+R_{f}}$$

After DG1 integration

(7) $$I_{1}^{\prime }=\frac{E_{s}+I_{2}^{\prime }\cdot R_{f}}{Z_{s}+Z_{\mathrm{AB}}+\beta Z_{\mathrm{BC}}+R_{f}}$$

After DG2 integration

(8) $$I_{1}^{\prime }=\frac{E_{s}-I_{2}^{\prime }\cdot R_{f}}{Z_{s}+Z_{\mathrm{AB}}+\beta Z_{\mathrm{BC}}+R_{f}}$$

Before DG integration, $I_{2}\approx 0$, ${I^{\prime }}_{2}>I_{2}$, from Equations (7) and (8), results show that DG connected upstream of the fault zone raises fault currents at QF1 and QF2. In contrast, DG connected downstream of the fault zone reduces fault currents at these two breakers.

### 2.3. Effects of DG Location, Capacity and Quantity on Fault Current

After short-circuit faults, fault signals change with DG location, capacity, and quantity. This paper analyzes zero-sequence current in detail. DG capacity is adjusted by changing the equivalent impedance in the simulation.

**Figure 4:**
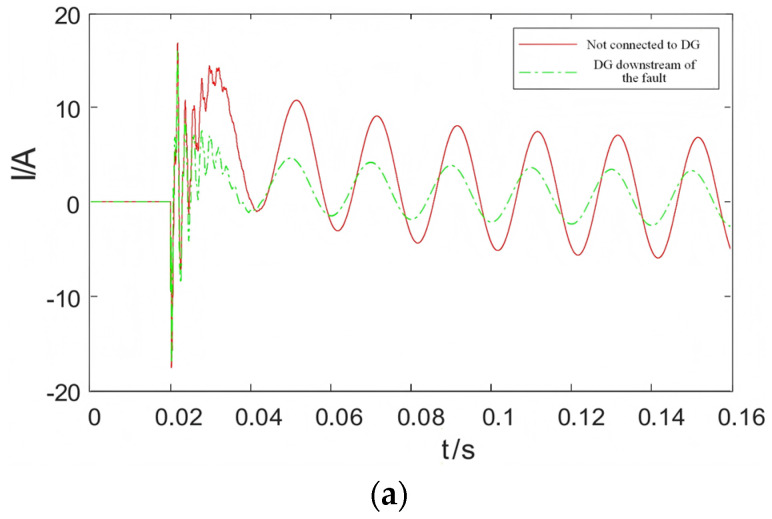

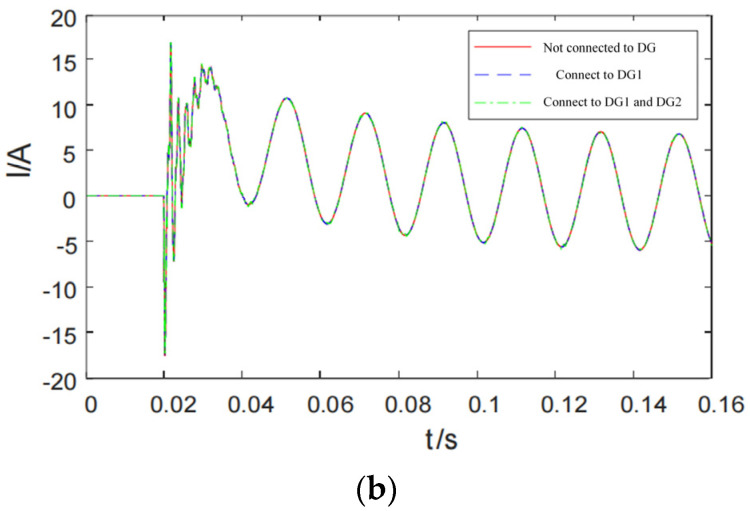
Zero-sequence current at the beginning of fault line L4. (**a**) Change in the access position of DG. (**b**) Changes in the number and capacity of DG connections.

Three cases are designed: no DG, DG upstream, and DG downstream. The inverter-type DG is set to 1 MW. Another test uses no DG, single DG (1 MW), and double DGs (1 MW + 2 MW). An A-phase ground fault is set on L4 at 2 km from the bus, with the fault time t = 0.02 s. The simulation results are shown in Figure 4.

Simulation outcomes indicate that DG integration, particularly downstream access, considerably alters both the direction and magnitude of zero-sequence current. The forward zero-sequence current supplied by the system and the reverse current injected by the DG tend to offset each other. This reduces the zero-sequence current amplitude at the source side. For a single DG, the current amplitude does not obviously rise with capacity growth. When multiple DGs are connected, the grid becomes a multi-source system. Multiple DGs may inject current into the fault point at the same time. The phase and direction of zero-sequence current become irregular. Traditional methods based on zero-sequence current may fail.

### 2.4. Fault Feature Extraction Using Wavelet Packet Transform

The wavelet packet transform is capable of decomposing both low-frequency and high-frequency elements of signals. It fully describes the time-frequency information of signals. For signal $X\left(n\right)$ decomposed into K layers, the time–frequency matrix of the K-th layer sub- bands is as follows:

(9) $$H_{X(n)}=\left[\begin{array}{cccc} a_{\left(1,1\right)} & a_{\left(1,2\right)} & \ldots & a_{\left(1,n\right)}\\ a_{\left(2,1\right)} & a_{\left(2,2\right)} & \ldots & a_{\left(2,n\right)}\\ \vdots & \vdots & \ddots & \vdots \\ a_{\left(2^{\kappa },1\right)} & a_{\left(2^{\kappa },2\right)} & \ldots & a_{\left(2^{\kappa },n\right)} \end{array}\right]$$

where $H_{X(n)}$ is the time–frequency matrix of the signal X(n); n is the number of signal sampling points; K is the number of layers of the wavelet packet transform, a natural number from 0 to K; $a_{\left(2^{K},n\right)}$ is the value of the $2^{K}$ sub-band in the K-th layer at the n-th sampling point.

The time–frequency matrices of the three-phase voltages, three-phase currents, and zero-sequence voltage are combined from top to bottom into a new time–frequency matrix as follows:

(10) $$W={\left[H_{U_{A}}^{T}H_{U_{B}}^{T}H_{U_{C}}^{T}H_{I_{A}}^{T}H_{I_{B}}^{T}H_{I_{C}}^{T}H_{U_{0}}^{T}\right]}^{T}$$

where $U_{A}$, $U_{B}$, $U_{C}$ are the three-phase voltages; $I_{A}$, $I_{B}$, $I_{C}$ are the three-phase currents; $U_{0}$ is the zero-sequence voltage; $H_{U_{A}}$, $H_{U_{B}}$, $H_{U_{C}}$ are the time–frequency matrices formed by the three-phase voltages, respectively; $H_{I_{A}}$, $H_{I_{B}}$, $H_{I_{C}}$ are the time–frequency matrices formed by the three-phase currents, respectively; $H_{U_{0}}$ is the time–frequency matrix formed by the zero-sequence voltage.

To further enhance the ability of the time–frequency matrix to represent the differences between signals and to improve the computational efficiency of the network, the time–frequency matrix is first taken as absolute values and then normalized.

(11) $$G=\frac{\left|W\right|}{\omega_{\max}}$$

where G is the time–frequency matrix obtained by taking the absolute value of W and then normalizing it; $\omega_{\max}$ is the maximum value in $\left|W\right|$.

The matrix G in Equation (11) is the finally obtained time–frequency matrix, and converting the time–frequency matrix into the form of a time-frequency image can serve as the input sample for the improved CNN-BiGRU model.

### 2.5. Generation of Time-Frequency Images from Fault Electrical Signal Samples

The sampling frequency is configured as 10 kHz. Signal samples are gathered from one cycle prior to the fault occurrence up to two cycles after the fault. The db4 wavelet is used as the basis function. Four-layer decomposition produces 16 frequency-band signals. Sub-bands are sorted from low to high center frequency.

**Figure 5:**
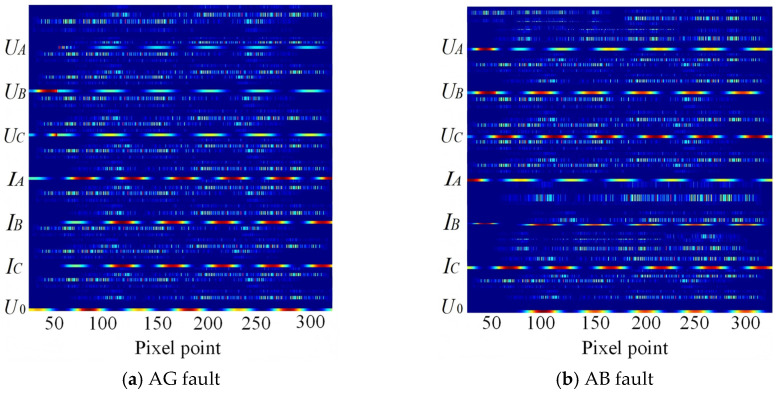

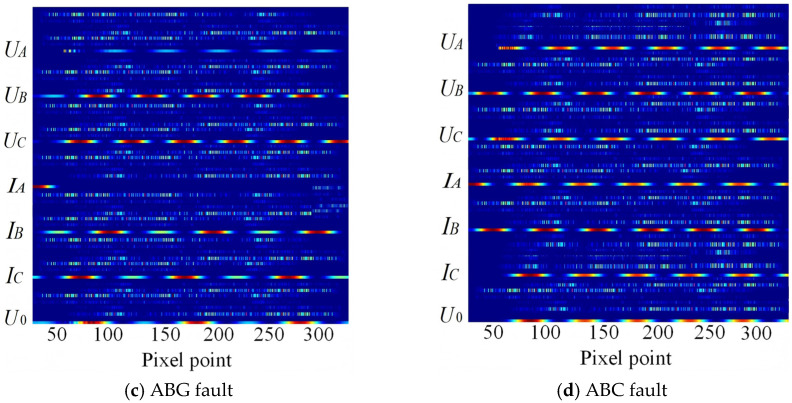
Time-frequency diagram of a power signal fault.

The time–frequency matrix is a pure data matrix. The wcodemat function in MATLAB maps its value range to [0, 255]. It turns the data matrix into a pixel matrix with image features. Each pixel’s color reflects its numerical size. Time-frequency images contain both low-frequency main information and high-frequency mutation details. Different fault types show different textures and color distributions. Four typical fault images are shown in Figure 5.

After implementing the wavelet packet transform (WPT) on three types of fault electrical signals, namely three-phase voltage, three-phase current and zero-sequence voltage, the corresponding time-frequency distribution diagrams are presented in Figure 5. In these time-frequency representations, the regions with relatively large amplitudes correspond to low-frequency components, which carry the main characteristic information of the fault signals. In contrast, the regions with smaller amplitudes are associated with high-frequency components, which characterize the transient mutation information of the signals during faults. It can be clearly seen that the wavelet packet time-frequency spectrogram is able to comprehensively extract and describe the full time-frequency features of fault electrical signals, which conforms to the signal analysis principles adopted in power system fault diagnosis research.

## 3. Fault Type Identification Model for Distribution Networks with DG Based on Improved CNN-BiGRU

**Figure 6:**
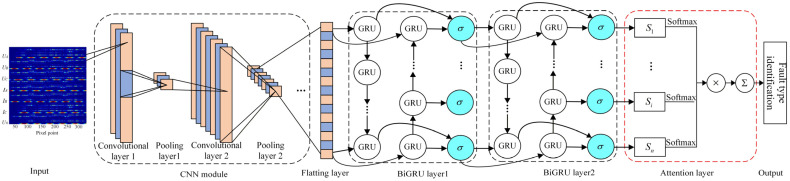
CNN-BiGRU-Attention model architecture diagram.

The time-frequency images obtained for the distribution network with DG under different fault types are different, and taking them as feature identification quantities, a fault identification model for distribution networks with DG based on improved CNN-BiGRU is proposed, as shown in Figure 6.

### 3.1. Convolutional Neural Network (CNN)

CNN consists of an input layer, convolutional layers, pooling layers, fully connected layers, and an output layer. Convolutional layers are responsible for extracting spatial features from the input data, while pooling layers filter redundant information and reduce the number of model parameters to prevent overfitting.

Convolution operation:

(12) $$x_{j}^{l}=f(\sum (x_{i}^{l-1}\cdot \omega_{ij}^{l})+b_{j}^{l})$$

where $x_{j}^{l}$ is the j-th feature map of the l-th layer; $x_{i}^{l-1}$ is the i-th feature map of the previous layer; $\omega_{ij}^{l}$ is the weight matrix of the l-th layer; $b_{j}^{l}$ is the corresponding bias term; f(⋅) is the activation function, usually the ReLU function.

Pooling operation:

(13) $$x_{j}^{l}=f(\beta_{j}^{l}\mathrm{down}(x_{j}^{l-1},M^{l})+b_{j}^{l})$$

where $\beta_{j}^{l}$ is the weight of the j-th feature map of the l-th layer; down(⋅) is the pooling function, including max pooling, average pooling, and random pooling, etc.; $M^{l}$ is the pooling window size $M^{l}\times M^{l}$ adopted by the l-th layer.

### 3.2. Bidirectional Gated Recurrent Unit (BiGRU)

GRU is a lightweight variant of the recurrent neural network (RNN). It employs update gates and reset gates to mitigate the problem of gradient vanishing. It has fewer parameters and faster convergence than LSTM. The calculation formulas are as follows:

(14) $$\left\{\begin{array}{l} z_{t}=\sigma \left(W_{z}\cdot \left[x\left(t\right),h\left(t-1\right)\right]\right)\\ r_{t}=\sigma \left(W_{r}\cdot \left[x\left(t\right),h\left(t-1\right)\right]\right)\\ \hat{h}\left(t\right)=\tanh\left(W_{h}\cdot \left[x\left(t\right),\left(r_{t}\cdot h\left(t-1\right)\right)\right]\right)\\ h\left(t\right)=\left(1-z_{t}\right)\cdot h\left(t-1\right)+z_{t}\cdot \hat{h}\left(t\right) \end{array}\right.$$

where σ(⋅) and tanh(⋅) are activation functions; x(t) is the output data of the previous state; $W_{z}$, $W_{r}$ and $W_{h}$ are the weight matrices of the update gate, reset gate, and candidate output, respectively.

**Figure 7:**
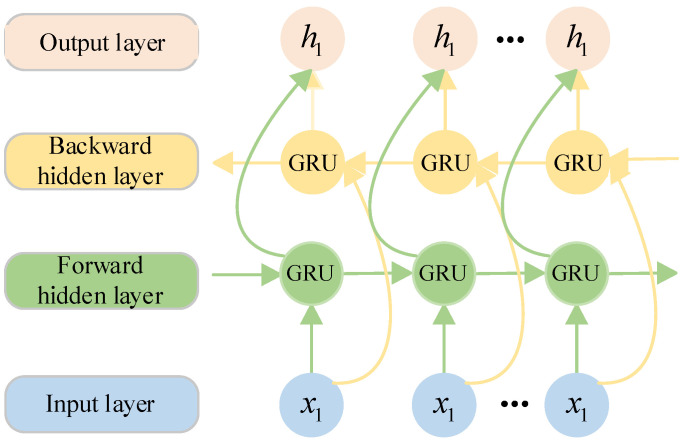
BiGRU structure.

BiGRU uses forward and backward GRU layers. It can capture time-dependent features missed by unidirectional GRU. It is very suitable for time–series fault signals. As shown in Figure 7.

### 3.3. Structure and Improvement of CNN-BiGRU

**Figure 8:**
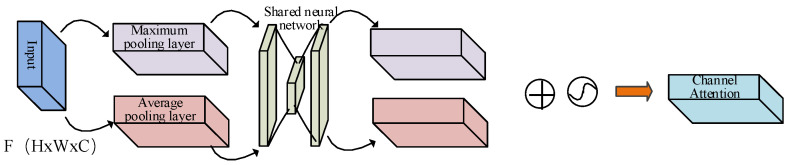
Channel attention structure diagram.

To further strengthen the feature extraction performance of the CNN-BiGRU framework when processing time-frequency images converted from fault electrical signals, an attention mechanism is introduced into the fully connected layer. Commonly applied in computer vision tasks, attention mechanisms assign adaptive weight coefficients to different input regions. This design enables the model to concentrate on key information that contributes more to fault recognition, thus enhancing model optimization and fault feature representation. Attention mechanisms encompass various types, such as channel attention, spatial attention, temporal attention, and other related variants. In this work, the channel attention module is adopted, and its computing flow is illustrated in Figure 8.

The input feature map, denoted as F (H × W × C), first goes through two parallel pooling operations. Global average pooling and global max pooling are adopted separately to capture representative fault characteristics. The resulting feature maps are then fed into a shared neural network. This network consists of a multi-layer perceptron with a single hidden layer, and is used to compute the final channel feature map. The mathematical formulation of the channel attention mechanism can be expressed as follows:

(15) $$\left\{\begin{array}{l} avgp=MLP(Avgp(F))\\ \max p=MLP(Maxp(F))\\ M_{c}(F)=\sigma (avgp+\max p) \end{array}\right.$$

where Avgp(F) represents average pooling; Maxp(F) represents max pooling; MLP( ) represents the multi-layer perceptron; σ represents the sigmoid activation function; $M_{c}(F)$ represents the channel attention matrix.

Apply the channel attention matrix to the original feature matrix:

(16) $$F^{\prime }=F⊗M_{c}(F)$$

The time-frequency images obtained from the wavelet packet transform are taken as 64 × 64 × 3 RGB images as the input of the CNN-BiGRU. After three convolutional pooling and three convolutional pooling activation units, the output is 2 × 2 × 256. After dual pooling, the output size becomes 1 × 1 × 256. The channel attention mechanism is added to weight the 256-dimensional channels. After the fully connected layer, the feature data are input into Softmax for classification to complete the identification result.

## 4. Fault Diagnosis Process

The whole identification process includes three steps:

(1) Step 1: Data preprocessing

The wavelet packet transform is applied to decompose three-phase voltage, three-phase current, and zero-sequence voltage signals. The first five frequency bands of the fourth decomposition layer are utilized to reconstruct time–frequency matrices, which are then converted into time-frequency images.

(2) Step 2: Model training

The dataset is divided into a training set, a validation set, and a test set. A CNN-BiGRU model integrated with a channel attention mechanism is constructed and trained accordingly.

(3) Step 3: Fault diagnosis

New signals undergo the same preprocessing procedure, which is then fed into the trained model. The model ultimately outputs the fault type.

**Figure 9:**
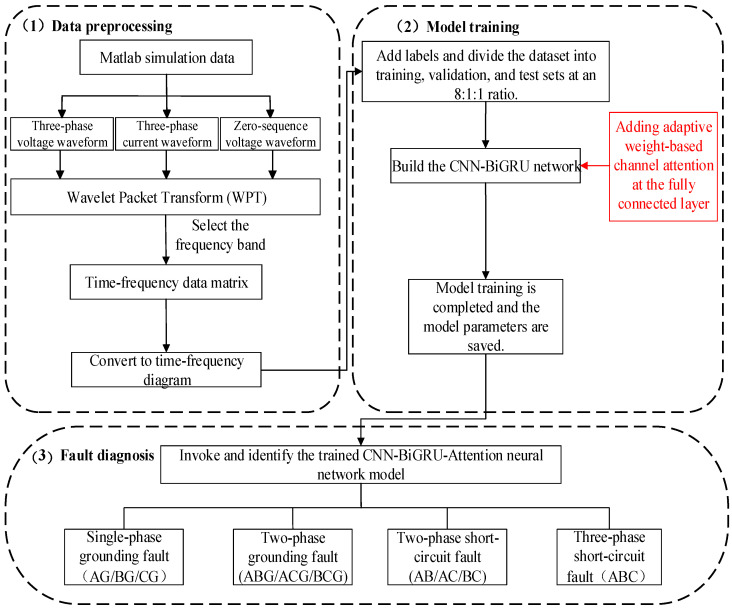
Fault identification flow chart.

The flow chart is illustrated in Figure 9.

## 5. Numerical Example Analysis

**Figure 10:**
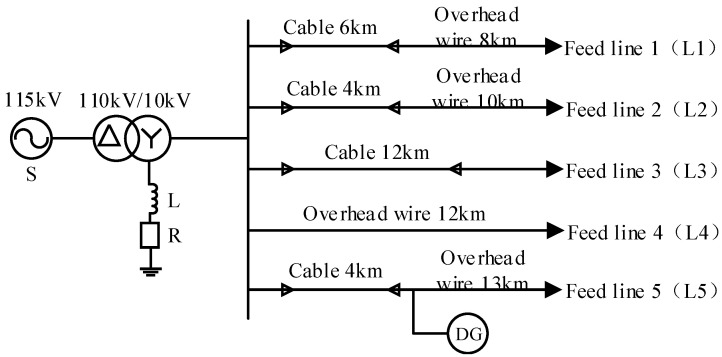
Schematic diagram of a 10kV distribution network with DG.

To verify the practicability and effectiveness of the proposed diagnosis scheme, this paper builds a 10 kV distribution network simulation model with distributed generation on the MATLAB/Simulink simulation platform, as shown in Figure 10. The system includes L1 to L5, a total of five feeders. Line parameters are listed in Table 1. The arc suppression coil uses 10% overcompensation, and the resistance is taken as 3% of the reactance value. After calculation, the inductance value is 0.8140 H, and the resistance is 8.2583 Ω. This paper takes the arc suppression coil grounding system as an example for analysis.

The sampling rate is set to 10 kHz. Ten types of faults are simulated, including single-phase grounding faults (AG, BG, CG), two-phase grounding faults (ABG, ACG, BCG), two-phase short-circuit faults (AB, AC, BC), and three-phase short-circuit faults (ABC).

### 5.1. Basis for Simulation Model Configuration and Parameter Selection

To clarify the rationality of the established MATLAB/Simulink model, the basis for the simulation structure and parameter selection is explained. A 10 kV distribution network with distributed generation is constructed because 10 kV systems are widely used in medium-voltage distribution networks and are representative for analyzing grounding faults and short-circuit faults. The model contains five feeders, L1–L5, including both overhead lines and cable lines. The positive-sequence and zero-sequence parameters of different line types are configured separately according to typical engineering parameters of 10 kV distribution lines, so that the influence of line impedance and line-to-ground capacitance on fault characteristics can be considered.

The neutral grounding mode is set as an arc suppression coil grounding system. This configuration is adopted because non-effectively grounded systems are common in medium-voltage distribution networks, and their grounding faults usually contain obvious transient components. The arc suppression coil adopts 10% overcompensation to avoid complete resonance compensation and to represent the practical condition in which the inductive compensation current is slightly larger than the capacitive current. The resistance of the arc suppression coil is set to 3% of its reactance to represent the active loss of the coil. According to the total line-to-ground capacitance and compensation degree, the inductance and resistance of the arc suppression coil are calculated as 0.8140 H and 8.2583 Ω, respectively.

The DG configuration is designed to analyze the influence of DG access location, capacity, and quantity on fault characteristics. After DG is connected, the distribution network changes from a single-source radial system to a multi-source active network. Therefore, different DG access positions are considered to represent upstream and downstream DG relative to the fault point. The upstream DG may produce a current boosting effect, while the downstream DG may produce a current drawing effect. In addition, different DG capacities and multiple DG access scenarios are set by changing the equivalent impedance of the DG model, so as to evaluate the adaptability of the proposed method under different DG penetration levels.

Ten fault categories are simulated, including single-phase grounding faults, two-phase grounding faults, two-phase short-circuit faults, and three-phase short-circuit faults. These fault types cover the main asymmetric and symmetric faults in distribution networks. The fault occurrence time is set after the system reaches a stable operating state. The sampling frequency is set to 10 kHz to capture high-frequency transient components. For each sample, the data window contains one cycle before the fault and two cycles after the fault, which provides both pre-fault steady-state information and post-fault transient information for wavelet packet decomposition and time-frequency image construction.

In the fault simulation, a detailed dynamic arc model, such as the Cassie, Mayr, or Cassie–Mayr model, is not adopted. The fault path is represented by an equivalent transition resistance to describe the impedance characteristics between the fault point and ground or between faulted phases. Therefore, the simulation mainly considers the influence of fault type and transition resistance on voltage and current transient waveforms. Since the dynamic variation in arc conductance may further affect high-frequency transient components, more detailed arc models will be introduced in future work to evaluate the robustness of the proposed method under realistic arcing fault conditions.

### 5.2. Fault Type Identification Test and Comparative Analysis of Distribution Network with DG

The configurations of the experimental platform are shown in Table 2.

The dataset is divided into a training set, a validation set, and a test set. In this study, ten fault categories are considered, including AG, BG, CG, ABG, ACG, BCG, AB, AC, BC, and ABC faults. For each fault type, 4500 samples are generated under different fault conditions, including different DG access positions, DG capacities, fault locations, transition resistances, and fault inception angles. The samples are divided according to the ratio of 8:1:1. Among them, 3600 samples are used as the training set, 450 samples are used as the validation set, and 450 samples are used as the test set. The training set is used to update the parameters of the CNN-BiGRU-Attention model, the validation set is used to monitor the convergence process and adjust the model parameters, and the test set is used to evaluate the final fault identification performance. To avoid the influence of sample imbalance on classification results, the samples of each fault type are evenly distributed in the training, validation, and test sets.

**Figure 11:**
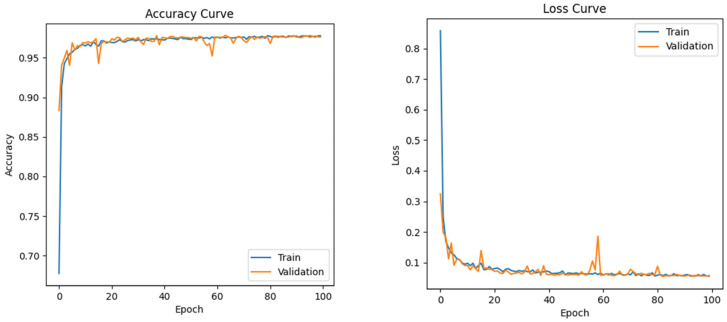
Comparison of accuracy and loss rate.

The accuracy and loss of the CNN-BiGRU-Attention fusion model during the training process on the training set are illustrated in Figure 11.

During network training, the Adam optimizer is employed for parameter updating. The batch size is configured as 50, the initial learning rate is set to 0.001, and the decay factor is 0.9. The model is trained for 100 epochs. The overall learning procedure for the 10 fault categories is displayed in Figure 11. It can be observed from the figure that the training loss declines steadily as the iteration increases and eventually converges to a stable level. Meanwhile, the classification accuracy increases continuously and gradually converges to a stable level. This outcome demonstrates that the presented CNN-BiGRU- Attention approach enjoys strong stability, rapid convergence, and high identification precision, and can effectively capture fault-related features. In addition, the training and validation curves show similar convergence trends, and no obvious divergence between training and validation performance is observed, indicating that no significant overfitting occurs under the current dataset division.

**Figure 12:**
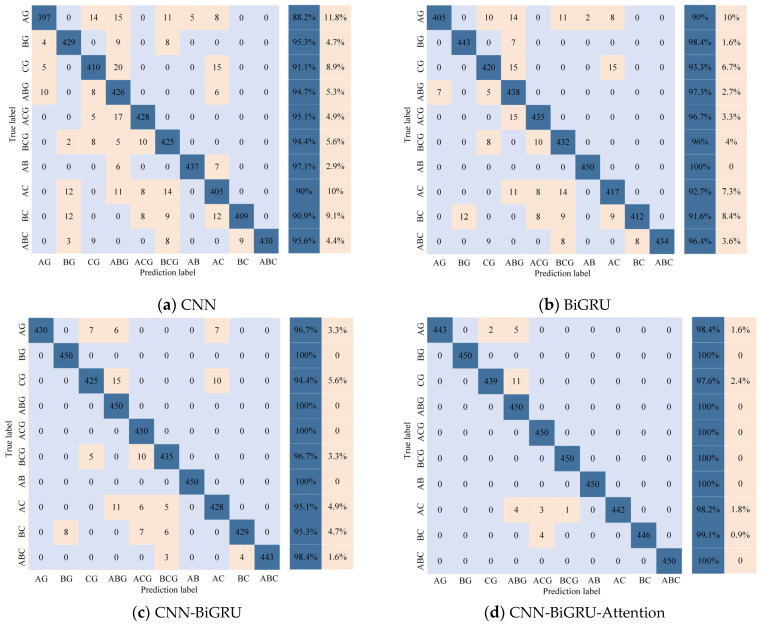
Experimental results of confusion matrices under different pattern recognition methods.

To clearly compare the fault recognition performance of different models, confusion matrices are used to analyze the classification results of the ten typical fault types in detail, as illustrated in Figure 12. In these matrices, elements on the main diagonal denote correctly identified samples, while the pink regions indicate misclassified samples. For the ten fault categories, the CNN method yields misjudgments on all types and achieves an overall accuracy of 91.55%. The BiGRU approach misjudges nine fault types; only the AB fault reaches 100% accuracy, with a total recognition rate of 94.71%. The CNN-BiGRU model produces seven types of misjudgments, and the accuracies of BG, ACG, and AB faults reach 100%, giving a comprehensive accuracy of 96.1%. In contrast, the proposed CNN-BiGRU-Attention method significantly reduces the number of misclassified samples, and its overall identification accuracy reaches 98.33%. The results verify that CNN-BiGRU-Attention provides the best classification performance. The main reason is that the hybrid CNN-BiGRU structure can extract more refined local spatial features from time-frequency maps of fault signals than single time-frequency analysis methods. 

### 5.3. Analysis of Model Adaptability and Generalization Ability

The t-Distributed Stochastic Neighbor Embedding (t-SNE) is a probability-driven nonlinear dimensionality reduction algorithm. After feature extraction, the dimensionality of the data samples is reduced, and high-dimensional data can be reduced to two-dimensional or three-dimensional space while preserving the local characteristics of the dataset. In the high-dimensional space, the local and global structures of the data are preserved by defining the samples using a Gaussian kernel function. The horizontal and vertical coordinates of the high-dimensional data points represent the first and second dimensional coordinates, respectively. In the t-SNE visualization, better classification performance is indicated by more compact distributions within the same fault category and clearer separation among different fault categories.

**Figure 13:**
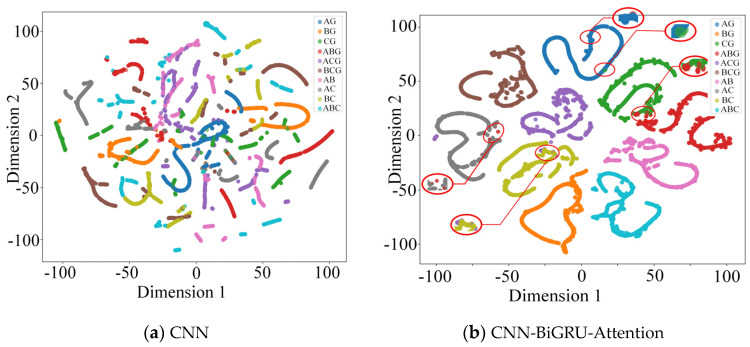
Without DG.

**Figure 14:**
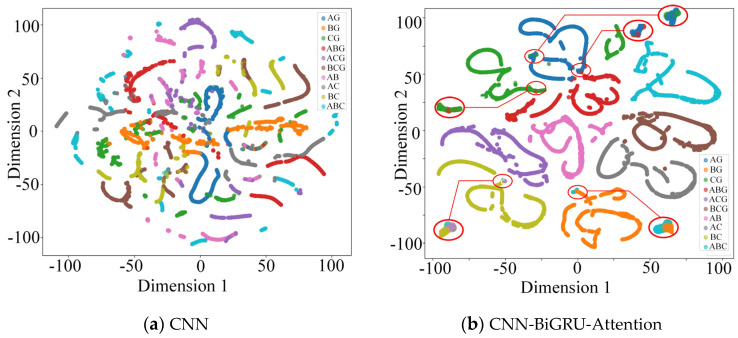
DG integrated.

By comparing Figure 13a,b and Figure 14a,b, it can be seen that after classification and identification by CNN-BiGRU-Attention, the various fault data present independent and clear clustering states. This indicates that the method has the best identification effect for the ten fault types and can fully extract fault features. According to the visualization diagram analysis, in the case without DG, five obvious fault type confusions occur, among which the main ones are as follows: AG faults confused as ABG and CG faults; CG faults confused as ABG faults; AC faults confused as ABG faults; and BC faults confused as BCG faults. The overall fault identification accuracy is 98.55%. In Figure 14b, when DG is added to the distribution network, six fault type confusions occur, and the accuracy of the confusion also relatively decreases, where AG faults are confused as CG and ABG faults; CG faults are confused as ABG faults; BC faults are confused as ABC and ACG faults; and BG faults are confused as ABC faults.

From Table 3, the rows correspond to the addition of CNN, BiGRU, CNN-BiGRU, and CNN-BiGRU-Attention, respectively.

The quantitative comparison in Table 3 further illustrates the limitations of single-structure models under DG-integrated conditions. When DG is integrated, the recognition accuracy of the single CNN model is 91.55%, and that of the single BiGRU model is 94.71%. After combining CNN and BiGRU, the accuracy increases to 96.14%, indicating that the joint extraction of spatial features and sequential features is beneficial for fault type identification. After introducing the channel attention mechanism, the accuracy further increases to 98.33%, which is 6.78%, 3.62%, and 2.19% higher than those of CNN, BiGRU, and CNN-BiGRU, respectively.

This result indicates that although existing time-frequency or deep learning methods can extract transient fault information, their feature representation ability may still be insufficient when DG changes the fault current magnitude, direction, and phase relationship. The proposed CNN-BiGRU-Attention model improves the identification performance by combining local spatial feature extraction, bidirectional temporal feature learning, and adaptive channel weighting. Therefore, the limitation of existing approaches in this paper does not mean that they cannot process high-frequency transient components, but that their adaptability may decrease under complex DG-integrated scenarios with weak and coupled fault features.

### 5.4. Influence of Different Capacities and Different Numbers on Fault Identification

In the previous section, distribution network models without DG and with DG1 and DG2 were used. To further test its generalization ability to the integration of distributed generation, distributed generation DG3-DG5 are added to the model, with DG3 and DG4 being 0.5 MW and DG5 being 1 MW.

With the increase in the number of distributed generation systems integrated, the fault type identification accuracy is shown in Table 4. It can be seen that when a single DG is added, the average fault detection accuracy is 98.33%, which is slightly lower than that without DG, but the accuracy can still reach above 98%; during the process of increasing the number of DGs from 1 to 3, the fault detection accuracy decreases from 98.33% to 97.32%; and during the process of increasing the number of DGs from 3 to 5, the fault identification accuracy stabilizes at around 97%.

It should be noted that the DG model used in this study is an equivalent model. The current simulations mainly verify the adaptability of the proposed method under different DG access positions, capacities, and quantities. In practical distribution networks, different DG types, such as inverter-based photovoltaic generation, wind power generation, and synchronous generators, may show different fault responses due to their control strategies, current-limiting mechanisms, and transient characteristics. Therefore, the current results cannot fully represent all DG technologies.

However, the proposed method uses three-phase voltage, three-phase current, and zero-sequence voltage to construct time-frequency images through wavelet packet transform, rather than relying on a single steady-state feature or fixed threshold. This enables the model to learn richer fault information from both low-frequency and high-frequency components. The tests under different DG capacities and multiple DG access scenarios partially verify the adaptability of the method. In future work, fault samples from photovoltaic inverter models, wind turbine generator models, and synchronous generator models will be further included to improve the generalization ability of the trained model in real distribution networks.

### 5.5. Analysis of Computational Cost and Practical Applicability

To further evaluate the practical applicability of the proposed method, the computational cost is analyzed together with the identification results. The proposed fault identification process mainly includes two stages: offline model training and online fault identification. In the offline stage, the wavelet packet transform is used to construct time-frequency images, and the CNN-BiGRU-Attention model is trained using the generated fault samples. This stage requires relatively high computational resources because convolutional feature extraction, bidirectional recurrent learning, and attention-based feature weighting are jointly optimized. However, offline training does not directly affect the real-time fault identification process.

In the online stage, the trained model only needs to perform signal preprocessing, time-frequency image construction, and forward inference. Therefore, the online computational burden is much lower than that of the training stage. In this study, the model training time is approximately 3.5 h, the model size is 29.8 MB, and the average inference time for one test sample is approximately 17.5 ms. These results indicate that although the proposed method introduces additional computational cost compared with single-structure models, the computational burden is acceptable for online fault type identification after offline training.

Combined with the recognition results in Table 3 and Table 4, the proposed method achieves an identification accuracy of 98.33% under DG-integrated conditions and maintains an accuracy above 97% under different DG access scenarios. This shows that the CNN-BiGRU-Attention model improves identification accuracy and adaptability while keeping the online inference time within an acceptable range. Therefore, the proposed method provides a reasonable balance between recognition performance, model complexity, and practical applicability.

## 6. Conclusions

To address the challenges in fault type classification arising from weak feature magnitudes and high-frequency transient components in distribution networks integrated with distributed generation (DG), this study proposes a fault identification method based on the CNN-BiGRU-Attention mechanism. A 10 kV distribution network model containing DG is constructed in MATLAB/Simulink to conduct simulation verification. The key conclusions are summarized as follows:

(1) The integration of DG into the distribution network leads to obvious changes in the zero-sequence current at the fault point, and the variation largely depends on the access position. When the access point is closer to the fault position, the impact on zero-sequence current becomes greater. In particular, if DG is connected downstream of the fault, the zero-sequence current will drop sharply. As DG capacity rises, the short-circuit current also increases slightly, but the growth range is not significant.
(2) The combined form of CNN and BiGRU is introduced, and an adaptive weight channel attention module is added to the fully connected layer of the CNN-BiGRU model to enhance its feature classification ability. Compared with the traditional CNN network, the identification accuracy is improved by 6.78%. Moreover, the classification effect is significantly improved. Compared with the traditional CNN model, the proposed CNN-BiGRU-Attention model significantly reduces the number of misclassified samples and improves the overall recognition accuracy.
(3) In the analysis of model adaptability and generalization, the proposed method maintains high recognition accuracy under different DG access scenarios. When a single DG is integrated, the identification accuracy reaches 98.33%. As the number of DGs increases, the accuracy decreases slightly but remains above 97%.

In summary, the proposed method can achieve high recognition accuracy under different DG access conditions. Although the CNN-BiGRU-Attention model introduces additional computational cost during offline training compared with a single CNN or BiGRU model, the trained model has an acceptable inference time for online fault type identification. Future work will further optimize the network structure to reduce model size and improve deployment efficiency.

## Figures and Tables

**Table 1:** Parameters of overhead lines and cable lines.

Parameter Types	*R*/(Ω/km)	*C*/(µF/km)	*L*/(mH/km)
Cable positive-sequence	0.031	0.338	0.096
Cable zero-sequence	0.234	0.265	0.355
Overhead line positive-sequence	0.178	0.125	0.098
Overhead line zero-sequence	0.251	0.056	0.456

**Table 2:** Configuration of the experimental platform.

Equipment	Configuration
System type	64-bit operating system, processor based on x64
Processor	12th Gen Intel(R) Core(TM) i5-12600KF@3.70 GHz
Graphics card	NVIDIA GeForce RTX 4060Ti
Operating system	Microsoft Windows11
Development language	Python

**Table 3:** Comparison of fault diagnosis algorithm accuracy effects.

Fault Diagnosis Algorithm	DG Integrated	Without DG
CNN	91.55%	92.41%
BiGRU	94.71%	95.40%
CNN-BiGRU	96.14%	96.57%
CNN-BiGRU-Attention	98.33%	98.55%

**Table 4:** Fault type identification accuracy after adding DG.

Distributed Power Supply Included	Accuracy
No DG	98.55%
DG1	98.33%
DG2	98.01%
DG1 + DG2	97.66%
DG1 + DG2 + DG3	97.32%
DG1 + DG2 + DG3 + DG4	97.11%
DG1 + DG2 + DG3 + DG4 + DG5	97.05%

## Data Availability

The data presented in this study are available on request from the corresponding author due to privacy.
